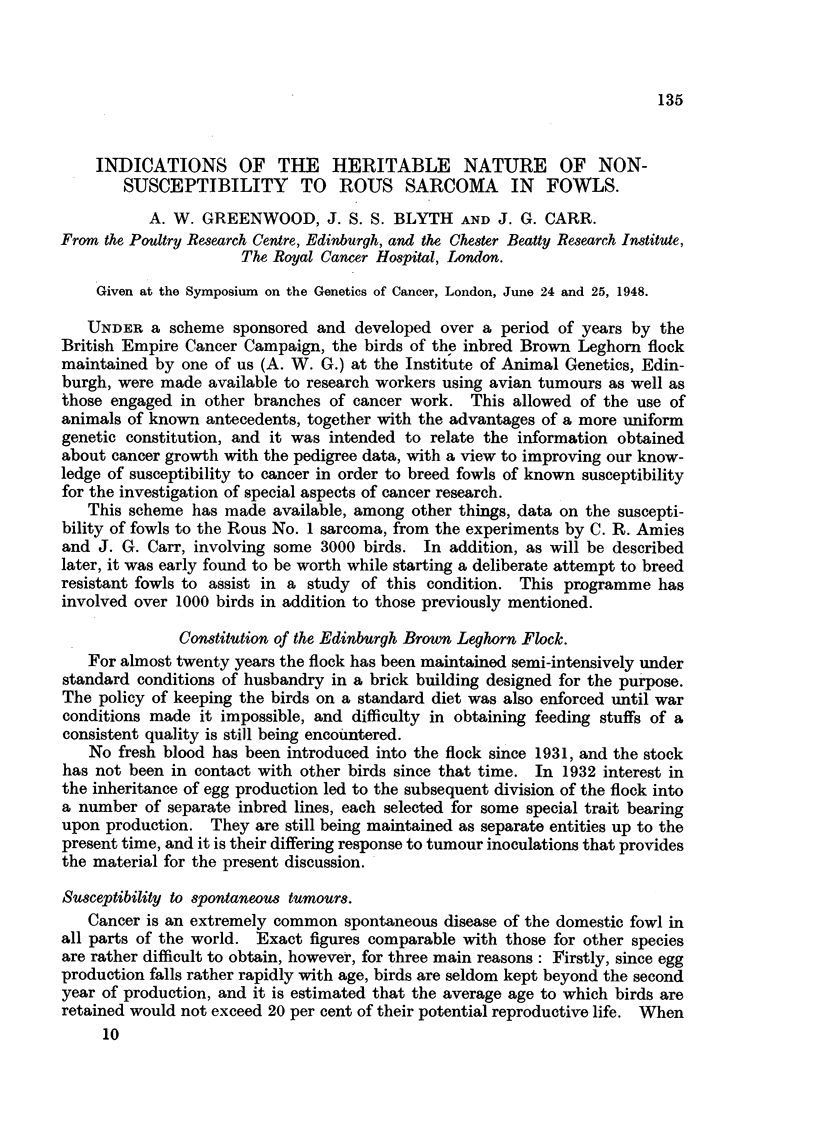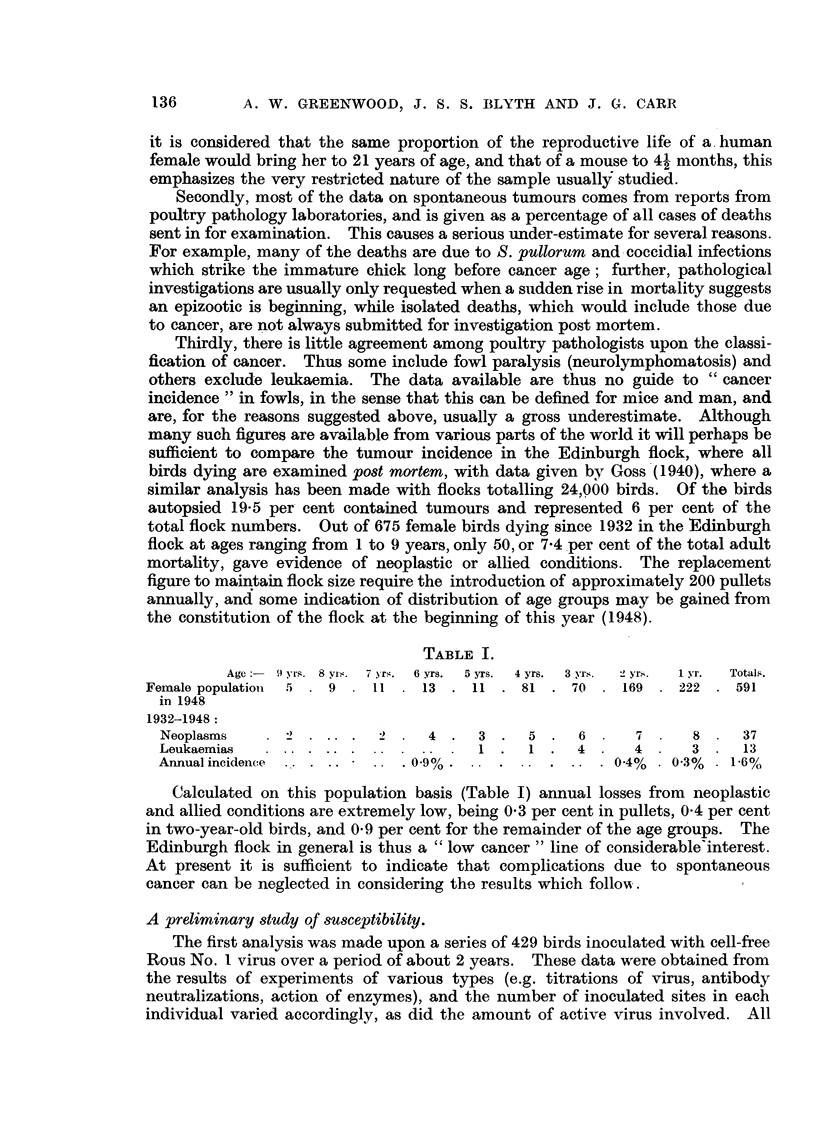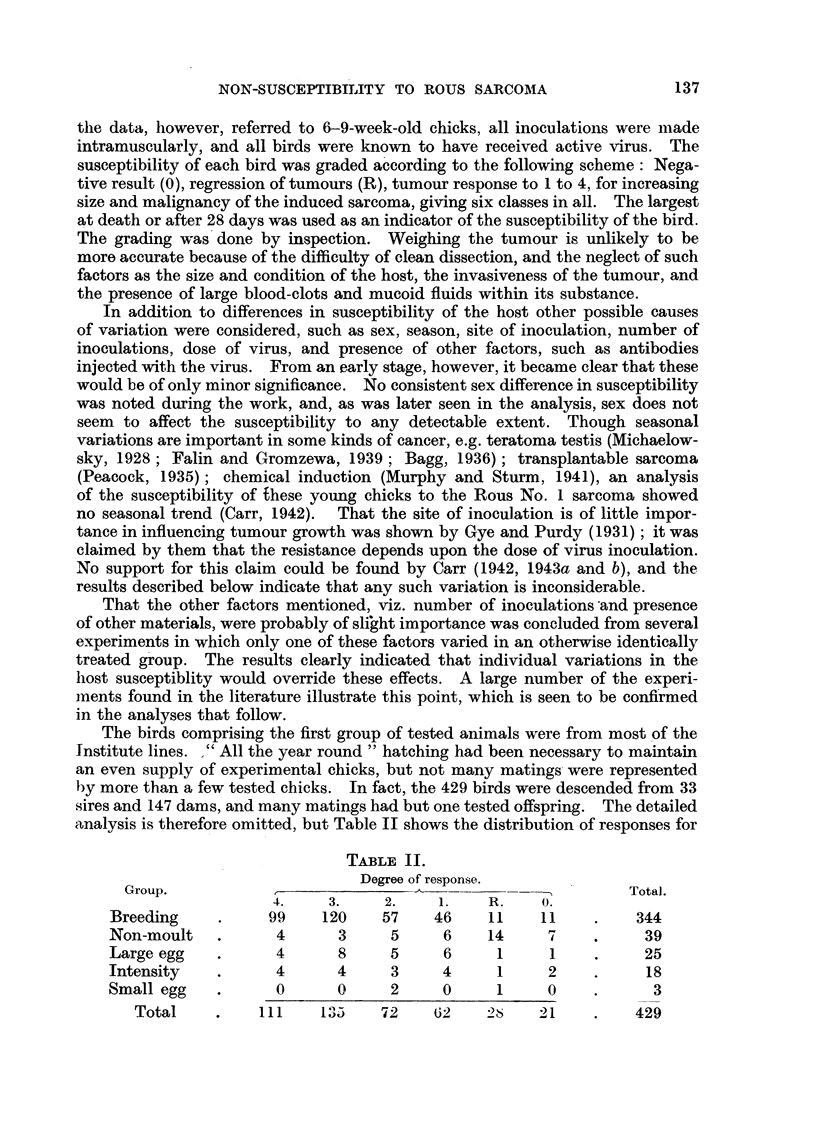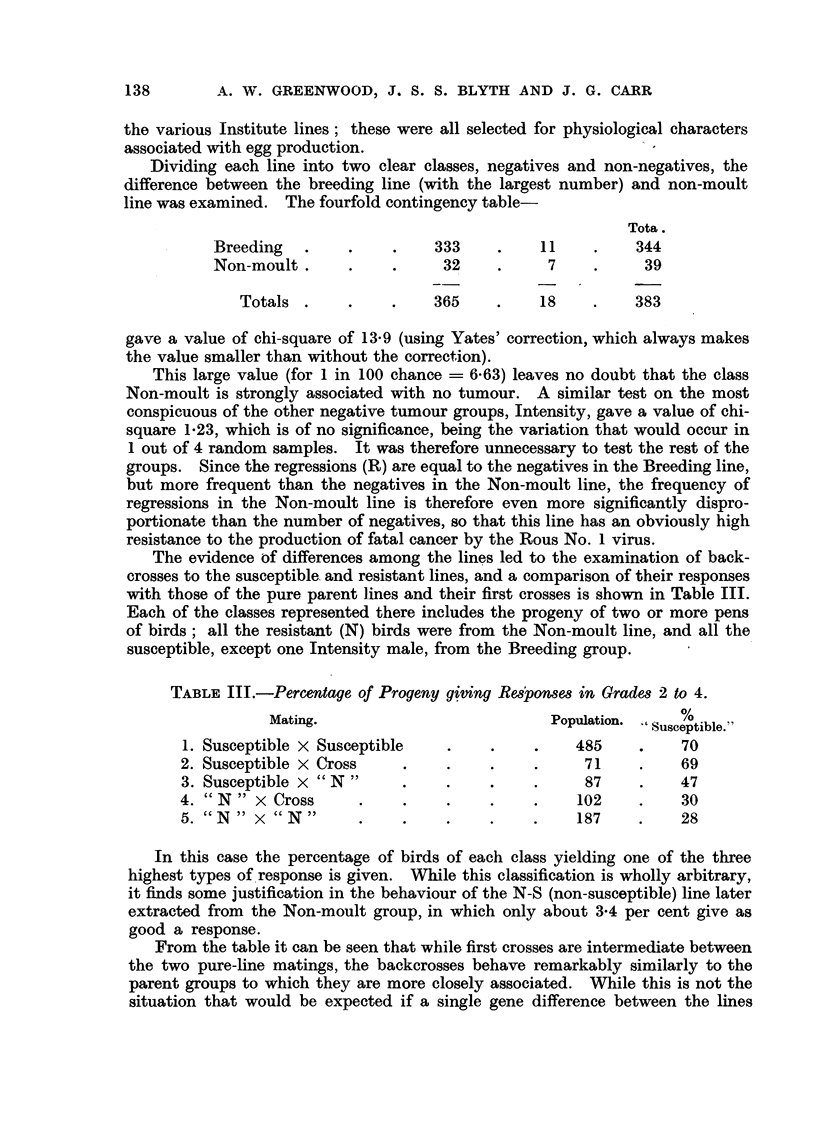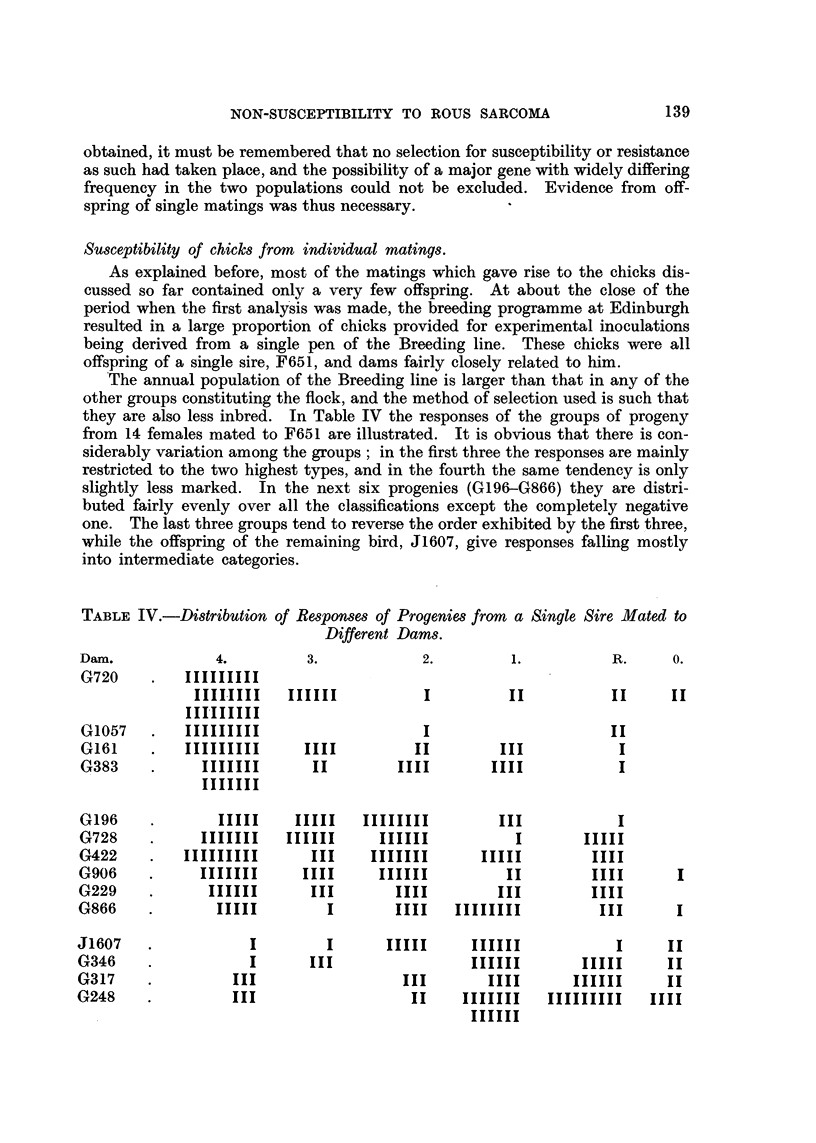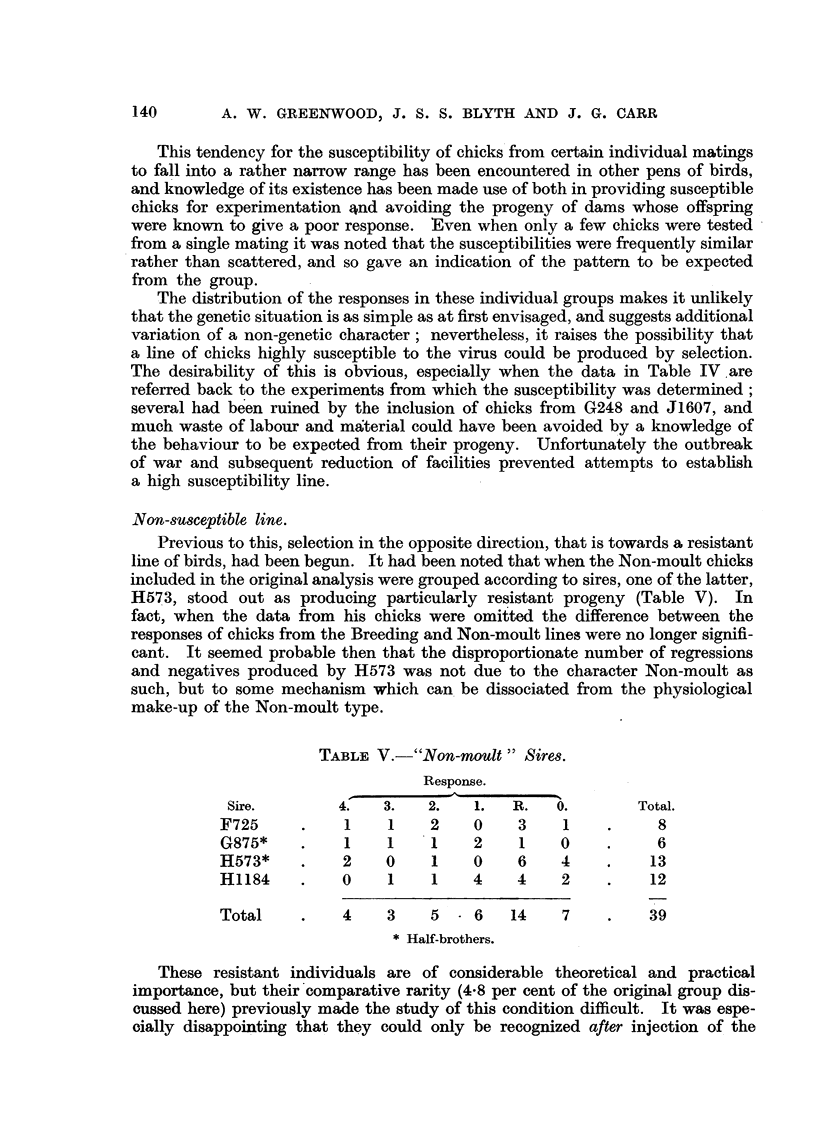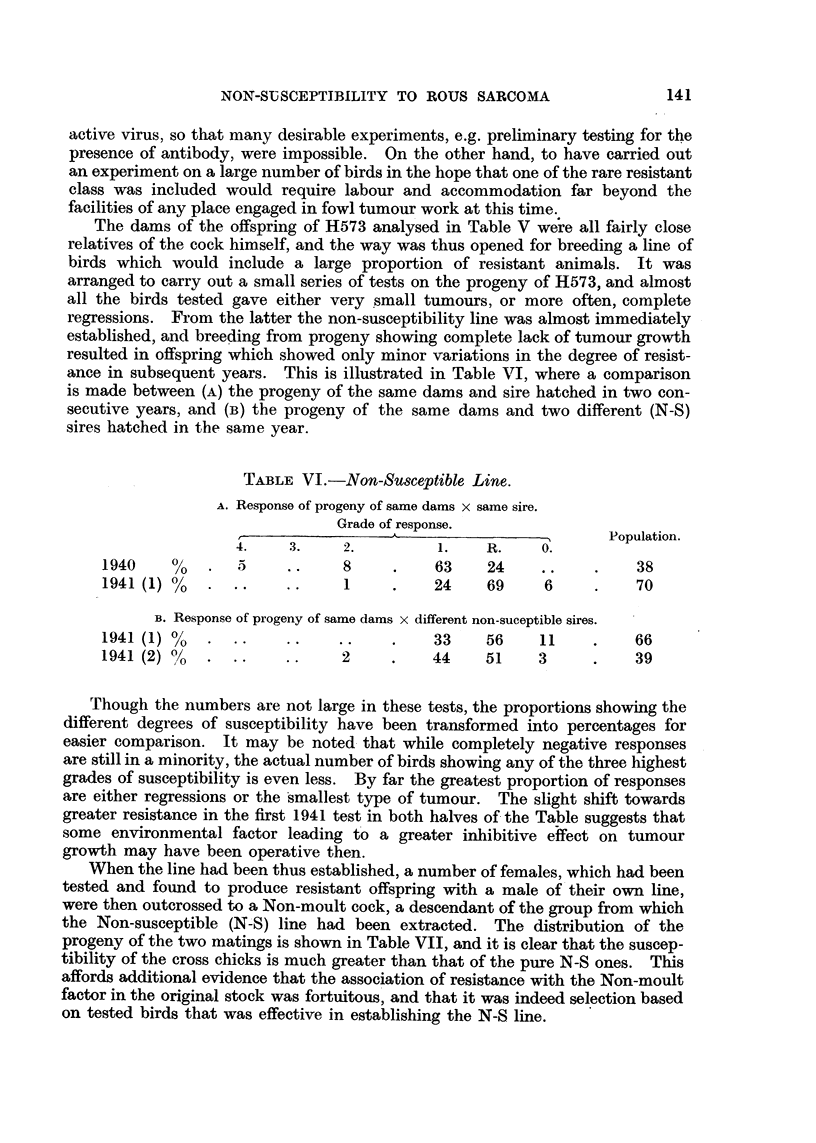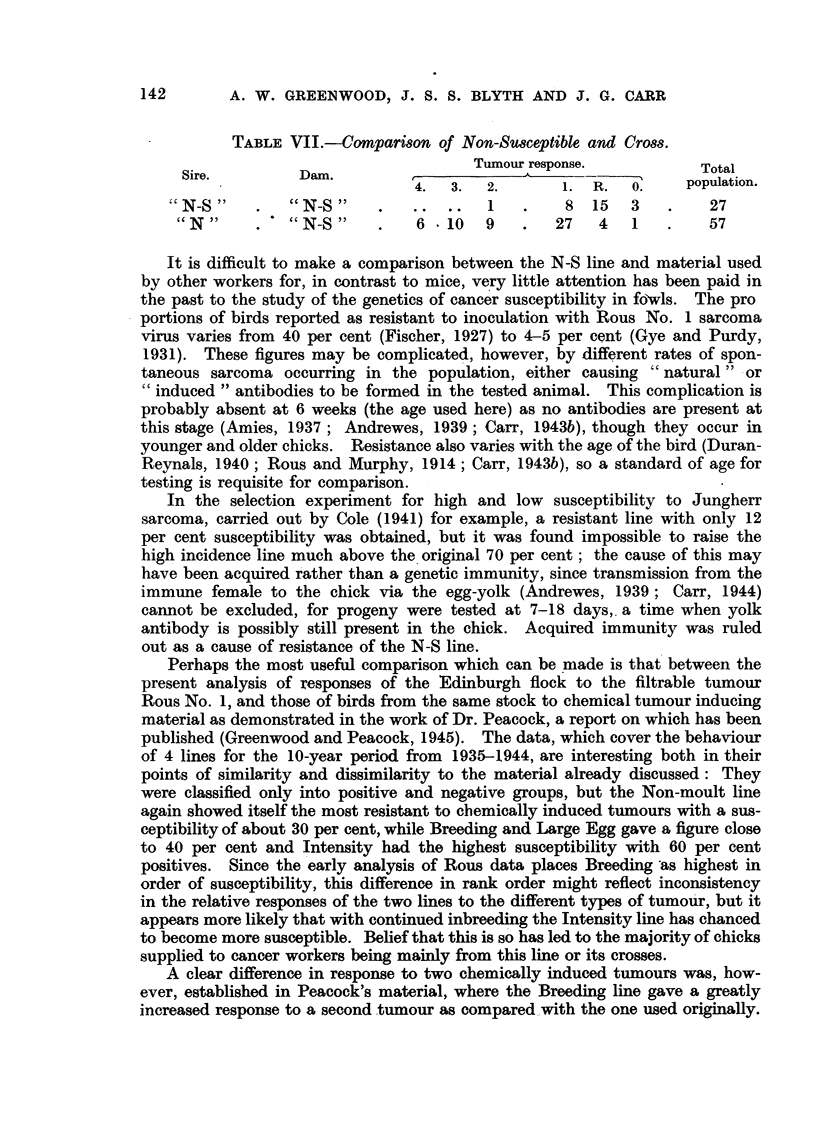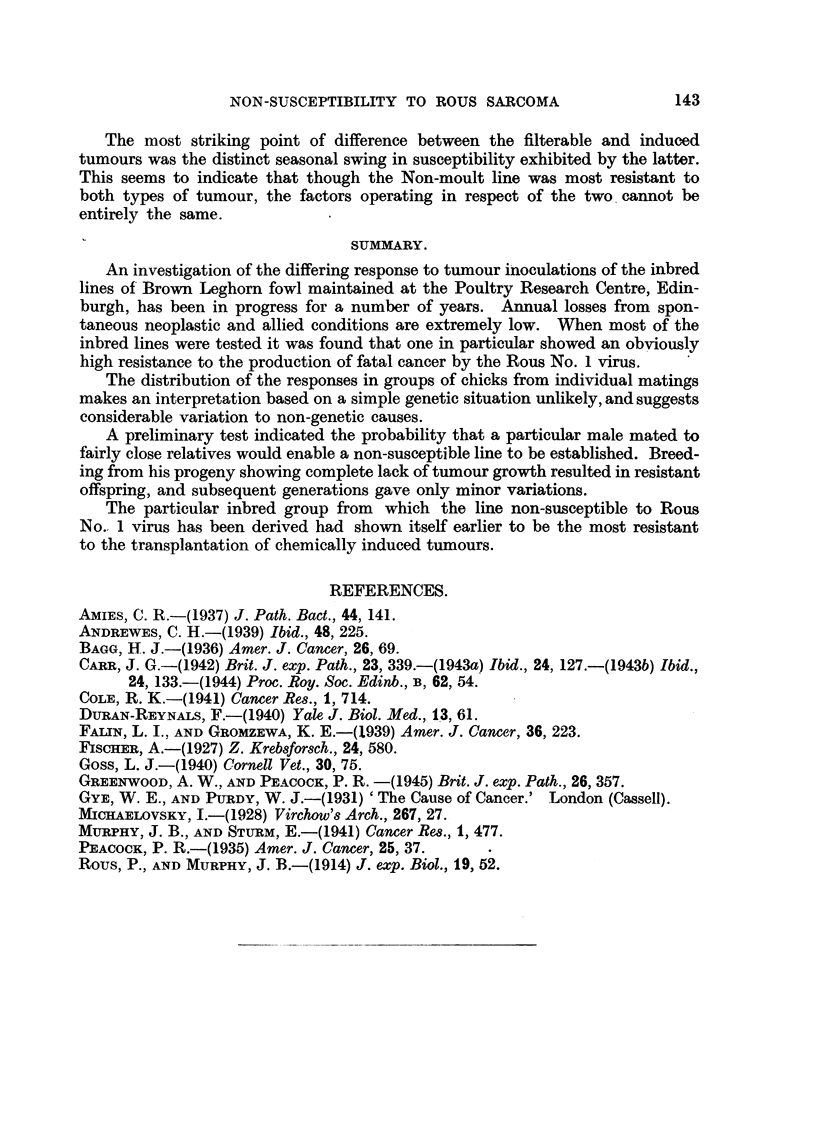# Indications of the Heritable Nature of Non-Susceptibility to Rous Sarcoma in Fowls

**DOI:** 10.1038/bjc.1948.20

**Published:** 1948-06

**Authors:** A. W. Greenwood, J. S. S. Blyth, J. G. Carr


### INDICATIONS OF THE HERITABLE NATURE OF NON-SUSCEPTIBILITY TO ROUS SARCOMA IN FOWLS.

A. W. GREENWOOD, J. S. S. BLYTH AND J. G. CARR.

*From the Poultry Research Centre, Edinburgh, and the Chester Beatty Research Institute, The Royal Cancer Hospital, London.*

Given at the Symposium on the Genetics of Cancer, London, June 24 and 25, 1948.

UNDER a scheme sponsored and developed over a period of years by the British Empire Cancer Campaign, the birds of the inbred Brown Leghorn flock maintained by one of us (A. W. G.) at the Institute of Animal Genetics, Edinburgh, were made available to research workers using avian tumours as well as those engaged in other branches of cancer work. This allowed of the use of animals of known antecedents, together with the advantages of a more uniform genetic constitution, and it was intended to relate the information obtained about cancer growth with the pedigree data, with a view to improving our knowledge of susceptibility to cancer in order to breed fowls of known susceptibility for the investigation of special aspects of cancer research.

This scheme has made available, among other things, data on the susceptibility of fowls to the Rous No. 1 sarcoma, from the experiments by C. R. Amies and J. G. Carr, involving some 3000 birds. In addition, as will be described later, it was early found to be worth while starting a deliberate attempt to breed resistant fowls to assist in a study of this condition. This programme has involved over 1000 birds in addition to those previously mentioned.

### *Constitution of the Edinburgh Brown Leghorn Flock.*

For almost twenty years the flock has been maintained semi-intensively under standard conditions of husbandry in a brick building designed for the purpose. The policy of keeping the birds on a standard diet was also enforced until war conditions made it impossible, and difficulty in obtaining feeding stuffs of a consistent quality is still being encountered.

No fresh blood has been introduced into the flock since 1931, and the stock has not been in contact with other birds since that time. In 1932 interest in the inheritance of egg production led to the subsequent division of the flock into a number of separate inbred lines, each selected for some special trait bearing upon production. They are still being maintained as separate entities up to the present time, and it is their differing response to tumour inoculations that provides the material for the present discussion.

### *Susceptibility to spontaneous tumours.*

Cancer is an extremely common spontaneous disease of the domestic fowl in all parts of the world. Exact figures comparable with those for other species are rather difficult to obtain, however, for three main reasons : Firstly, since egg production falls rather rapidly with age, birds are seldom kept beyond the second year of production, and it is estimated that the average age to which birds are retained would not exceed 20 per cent of their potential reproductive life. When it is considered that the same proportion of the reproductive life of a human female would bring her to 21 years of age, and that of a mouse to 4½ months, this emphasizes the very restricted nature of the sample usually studied.

Secondly, most of the data on spontaneous tumours comes from reports from poultry pathology laboratories, and is given as a percentage of all cases of deaths sent in for examination. This causes a serious under-estimate for several reasons. For example, many of the deaths are due to *S. pullorum* and coccidial infections which strike the immature chick long before cancer age ; further, pathological investigations are usually only requested when a sudden rise in mortality suggests an epizootic is beginning, while isolated deaths, which would include those due to cancer, are not always submitted for investigation post mortem.

Thirdly, there is little agreement among poultry pathologists upon the classification of cancer. Thus some include fowl paralysis (neurolymphomatosis) and others exclude leukaemia. The data available are thus no guide to " cancer incidence " in fowls, in the sense that this can be defined for mice and man, and are, for the reasons suggested above, usually a gross underestimate. Although many such figures are available from various parts of the world it will perhaps be sufficient to compare the tumour incidence in the Edinburgh flock, where all birds dying are examined *post mortem*, with data given by Goss (1940), where a similar analysis has been made with flocks totalling 24,000 birds. Of the birds autopsied 19·5 per cent contained tumours and represented 6 per cent of the total flock numbers. Out of 675 female birds dying since 1932 in the Edinburgh flock at ages ranging from 1 to 9 years, only 50, or 7·4 per cent of the total adult mortality, gave evidence of neoplastic or allied conditions. The replacement figure to maintain flock size require the introduction of approximately 200 pullets annually, and some indication of distribution of age groups may be gained from the constitution of the flock at the beginning of this year (1948).

TABLE I.

| Age :— | 9 yrs. | 8 yrs. | 7 yrs. | 6 yrs. | 5 yrs. | 4 yrs. | 3 yrs. | 2 yrs. | 1 yr. | Totals. |
|---|---|---|---|---|---|---|---|---|---|---|
| Female population in 1948 | 5 | 9 | 11 | 13 | 11 | 81 | 70 | 169 | 222 | 591 |
| 1932–1948 : | | | | | | | | | | |
| Neoplasms | 2 | .. | 2 | 4 | 3 | 5 | 6 | 7 | 8 | 37 |
| Leukaemias | .. | .. | .. | .. | 1 | 1 | 4 | 4 | 3 | 13 |
| Annual incidence | .. | .. | .. | 0·9% | .. | .. | .. | 0·4% | 0·3% | 1·6% |

Calculated on this population basis (Table I) annual losses from neoplastic and allied conditions are extremely low, being 0·3 per cent in pullets, 0·4 per cent in two-year-old birds, and 0·9 per cent for the remainder of the age groups. The Edinburgh flock in general is thus a " low cancer " line of considerable interest. At present it is sufficient to indicate that complications due to spontaneous cancer can be neglected in considering the results which follow.

*A preliminary study of susceptibility.*

The first analysis was made upon a series of 429 birds inoculated with cell-free Rous No. 1 virus over a period of about 2 years. These data were obtained from the results of experiments of various types (e.g. titrations of virus, antibody neutralizations, action of enzymes), and the number of inoculated sites in each individual varied accordingly, as did the amount of active virus involved. All the data, however, referred to 6–9-week-old chicks, all inoculations were made intramuscularly, and all birds were known to have received active virus. The susceptibility of each bird was graded according to the following scheme : Negative result (0), regression of tumours (R), tumour response to 1 to 4, for increasing size and malignancy of the induced sarcoma, giving six classes in all. The largest at death or after 28 days was used as an indicator of the susceptibility of the bird. The grading was done by inspection. Weighing the tumour is unlikely to be more accurate because of the difficulty of clean dissection, and the neglect of such factors as the size and condition of the host, the invasiveness of the tumour, and the presence of large blood-clots and mucoid fluids within its substance.

In addition to differences in susceptibility of the host other possible causes of variation were considered, such as sex, season, site of inoculation, number of inoculations, dose of virus, and presence of other factors, such as antibodies injected with the virus. From an early stage, however, it became clear that these would be of only minor significance. No consistent sex difference in susceptibility was noted during the work, and, as was later seen in the analysis, sex does not seem to affect the susceptibility to any detectable extent. Though seasonal variations are important in some kinds of cancer, e.g. teratoma testis (Michaelowsky, 1928 ; Falin and Gromzewa, 1939 ; Bagg, 1936) ; transplantable sarcoma (Peacock, 1935) ; chemical induction (Murphy and Sturm, 1941), an analysis of the susceptibility of these young chicks to the Rous No. 1 sarcoma showed no seasonal trend (Carr, 1942). That the site of inoculation is of little importance in influencing tumour growth was shown by Gye and Purdy (1931) ; it was claimed by them that the resistance depends upon the dose of virus inoculation. No support for this claim could be found by Carr (1942, 1943*a* and *b*), and the results described below indicate that any such variation is inconsiderable.

That the other factors mentioned, viz. number of inoculations and presence of other materials, were probably of slight importance was concluded from several experiments in which only one of these factors varied in an otherwise identically treated group. The results clearly indicated that individual variations in the host susceptiblity would override these effects. A large number of the experiments found in the literature illustrate this point, which is seen to be confirmed in the analyses that follow.

The birds comprising the first group of tested animals were from most of the Institute lines. " All the year round " hatching had been necessary to maintain an even supply of experimental chicks, but not many matings were represented by more than a few tested chicks. In fact, the 429 birds were descended from 33 sires and 147 dams, and many matings had but one tested offspring. The detailed analysis is therefore omitted, but Table II shows the distribution of responses for

TABLE II.

| Group. | Degree of response. 4. | 3. | 2. | 1. | R. | 0. | Total. |
|---|---|---|---|---|---|---|---|
| Breeding | 99 | 120 | 57 | 46 | 11 | 11 | 344 |
| Non-moult | 4 | 3 | 5 | 6 | 14 | 7 | 39 |
| Large egg | 4 | 8 | 5 | 6 | 1 | 1 | 25 |
| Intensity | 4 | 4 | 3 | 4 | 1 | 2 | 18 |
| Small egg | 0 | 0 | 2 | 0 | 1 | 0 | 3 |
| Total | 111 | 135 | 72 | 62 | 28 | 21 | 429 |

the various Institute lines; these were all selected for physiological characters associated with egg production.

Dividing each line into two clear classes, negatives and non-negatives, the difference between the breeding line (with the largest number) and non-moult line was examined. The fourfold contingency table—

| | | | Tota . |
|---|---|---|---|
| Breeding | 333 | 11 | 344 |
| Non-moult | 32 | 7 | 39 |
| Totals | 365 | 18 | 383 |

gave a value of chi-square of 13·9 (using Yates' correction, which always makes the value smaller than without the correction).

This large value (for 1 in 100 chance = 6·63) leaves no doubt that the class Non-moult is strongly associated with no tumour. A similar test on the most conspicuous of the other negative tumour groups, Intensity, gave a value of chi-square 1·23, which is of no significance, being the variation that would occur in 1 out of 4 random samples. It was therefore unnecessary to test the rest of the groups. Since the regressions (R) are equal to the negatives in the Breeding line, but more frequent than the negatives in the Non-moult line, the frequency of regressions in the Non-moult line is therefore even more significantly disproportionate than the number of negatives, so that this line has an obviously high resistance to the production of fatal cancer by the Rous No. 1 virus.

The evidence of differences among the lines led to the examination of backcrosses to the susceptible and resistant lines, and a comparison of their responses with those of the pure parent lines and their first crosses is shown in Table III. Each of the classes represented there includes the progeny of two or more pens of birds; all the resistant (N) birds were from the Non-moult line, and all the susceptible, except one Intensity male, from the Breeding group.

TABLE III.—*Percentage of Progeny giving Responses in Grades 2 to 4.*

| Mating. | Population. | % "Susceptible." |
|---|---|---|
| 1. Susceptible × Susceptible | 485 | 70 |
| 2. Susceptible × Cross | 71 | 69 |
| 3. Susceptible × "N" | 87 | 47 |
| 4. "N" × Cross | 102 | 30 |
| 5. "N" × "N" | 187 | 28 |

In this case the percentage of birds of each class yielding one of the three highest types of response is given. While this classification is wholly arbitrary, it finds some justification in the behaviour of the N-S (non-susceptible) line later extracted from the Non-moult group, in which only about 3·4 per cent give as good a response.

From the table it can be seen that while first crosses are intermediate between the two pure-line matings, the backcrosses behave remarkably similarly to the parent groups to which they are more closely associated. While this is not the situation that would be expected if a single gene difference between the lines obtained, it must be remembered that no selection for susceptibility or resistance as such had taken place, and the possibility of a major gene with widely differing frequency in the two populations could not be excluded. Evidence from offspring of single matings was thus necessary.

*Susceptibility of chicks from individual matings.*

As explained before, most of the matings which gave rise to the chicks discussed so far contained only a very few offspring. At about the close of the period when the first analysis was made, the breeding programme at Edinburgh resulted in a large proportion of chicks provided for experimental inoculations being derived from a single pen of the Breeding line. These chicks were all offspring of a single sire, F651, and dams fairly closely related to him.

The annual population of the Breeding line is larger than that in any of the other groups constituting the flock, and the method of selection used is such that they are also less inbred. In Table IV the responses of the groups of progeny from 14 females mated to F651 are illustrated. It is obvious that there is considerably variation among the groups ; in the first three the responses are mainly restricted to the two highest types, and in the fourth the same tendency is only slightly less marked. In the next six progenies (G196–G866) they are distributed fairly evenly over all the classifications except the completely negative one. The last three groups tend to reverse the order exhibited by the first three, while the offspring of the remaining bird, J1607, give responses falling mostly into intermediate categories.

TABLE IV.—*Distribution of Responses of Progenies from a Single Sire Mated to Different Dams.*

| Dam. | 4. | 3. | 2. | 1. | R. | 0. |
|---|---|---|---|---|---|---|
| G720 . | IIIIIIIII<br>IIIIIIII<br>IIIIIIIII | IIIIII | I | II | II | II |
| G1057 . | IIIIIIIII | | I | | II | |
| G161 . | IIIIIIIII | IIII | II | III | I | |
| G383 . | IIIIIII<br>IIIIIII | II | IIII | IIII | I | |
| G196 . | IIIII | IIIII | IIIIIIII | III | I | |
| G728 . | IIIIIII | IIIIII | IIIIII | I | IIIII | |
| G422 . | IIIIIIIII | III | IIIIIII | IIIII | IIII | |
| G906 . | IIIIIII | IIII | IIIIII | II | IIII | I |
| G229 . | IIIIII | III | IIII | III | IIII | |
| G866 . | IIIII | I | IIII | IIIIIIII | III | I |
| J1607 . | I | I | IIIII | IIIIII | I | II |
| G346 . | I | III | | IIIIII | IIIII | II |
| G317 . | III | | III | IIII | IIIIII | II |
| G248 . | III | | II | IIIIIII<br>IIIIII | IIIIIIIII | IIII |

This tendency for the susceptibility of chicks from certain individual matings to fall into a rather narrow range has been encountered in other pens of birds, and knowledge of its existence has been made use of both in providing susceptible chicks for experimentation and avoiding the progeny of dams whose offspring were known to give a poor response. Even when only a few chicks were tested from a single mating it was noted that the susceptibilities were frequently similar rather than scattered, and so gave an indication of the pattern to be expected from the group.

The distribution of the responses in these individual groups makes it unlikely that the genetic situation is as simple as at first envisaged, and suggests additional variation of a non-genetic character ; nevertheless, it raises the possibility that a line of chicks highly susceptible to the virus could be produced by selection. The desirability of this is obvious, especially when the data in Table IV are referred back to the experiments from which the susceptibility was determined ; several had been ruined by the inclusion of chicks from G248 and J1607, and much waste of labour and material could have been avoided by a knowledge of the behaviour to be expected from their progeny. Unfortunately the outbreak of war and subsequent reduction of facilities prevented attempts to establish a high susceptibility line.

*Non-susceptible line.*

Previous to this, selection in the opposite direction, that is towards a resistant line of birds, had been begun. It had been noted that when the Non-moult chicks included in the original analysis were grouped according to sires, one of the latter, H573, stood out as producing particularly resistant progeny (Table V). In fact, when the data from his chicks were omitted the difference between the responses of chicks from the Breeding and Non-moult lines were no longer significant. It seemed probable then that the disproportionate number of regressions and negatives produced by H573 was not due to the character Non-moult as such, but to some mechanism which can be dissociated from the physiological make-up of the Non-moult type.

TABLE V.—*"Non-moult" Sires.*

| Sire. | Response. 4. | 3. | 2. | 1. | R. | 0. | Total. |
|---|---|---|---|---|---|---|---|
| F725 | 1 | 1 | 2 | 0 | 3 | 1 | 8 |
| G875* | 1 | 1 | 1 | 2 | 1 | 0 | 6 |
| H573* | 2 | 0 | 1 | 0 | 6 | 4 | 13 |
| H1184 | 0 | 1 | 1 | 4 | 4 | 2 | 12 |
| Total | 4 | 3 | 5 | 6 | 14 | 7 | 39 |

* Half-brothers.

These resistant individuals are of considerable theoretical and practical importance, but their comparative rarity (4·8 per cent of the original group discussed here) previously made the study of this condition difficult. It was especially disappointing that they could only be recognized *after* injection of the active virus, so that many desirable experiments, e.g. preliminary testing for the presence of antibody, were impossible. On the other hand, to have carried out an experiment on a large number of birds in the hope that one of the rare resistant class was included would require labour and accommodation far beyond the facilities of any place engaged in fowl tumour work at this time.

The dams of the offspring of H573 analysed in Table V were all fairly close relatives of the cock himself, and the way was thus opened for breeding a line of birds which would include a large proportion of resistant animals. It was arranged to carry out a small series of tests on the progeny of H573, and almost all the birds tested gave either very small tumours, or more often, complete regressions. From the latter the non-susceptibility line was almost immediately established, and breeding from progeny showing complete lack of tumour growth resulted in offspring which showed only minor variations in the degree of resistance in subsequent years. This is illustrated in Table VI, where a comparison is made between (A) the progeny of the same dams and sire hatched in two consecutive years, and (B) the progeny of the same dams and two different (N-S) sires hatched in the same year.

TABLE VI.—*Non-Susceptible Line.*

A. Response of progeny of same dams × same sire.

| | Grade of response. 4. | 3. | 2. | 1. | R. | 0. | Population. |
|---|---|---|---|---|---|---|---|
| 1940 % | 5 | .. | 8 | 63 | 24 | .. | 38 |
| 1941 (1) % | .. | .. | 1 | 24 | 69 | 6 | 70 |

B. Response of progeny of same dams × different non-suceptible sires.

| | 4. | 3. | 2. | 1. | R. | 0. | Population. |
|---|---|---|---|---|---|---|---|
| 1941 (1) % | .. | .. | .. | 33 | 56 | 11 | 66 |
| 1941 (2) % | .. | .. | 2 | 44 | 51 | 3 | 39 |

Though the numbers are not large in these tests, the proportions showing the different degrees of susceptibility have been transformed into percentages for easier comparison. It may be noted that while completely negative responses are still in a minority, the actual number of birds showing any of the three highest grades of susceptibility is even less. By far the greatest proportion of responses are either regressions or the smallest type of tumour. The slight shift towards greater resistance in the first 1941 test in both halves of the Table suggests that some environmental factor leading to a greater inhibitive effect on tumour growth may have been operative then.

When the line had been thus established, a number of females, which had been tested and found to produce resistant offspring with a male of their own line, were then outcrossed to a Non-moult cock, a descendant of the group from which the Non-susceptible (N-S) line had been extracted. The distribution of the progeny of the two matings is shown in Table VII, and it is clear that the susceptibility of the cross chicks is much greater than that of the pure N-S ones. This affords additional evidence that the association of resistance with the Non-moult factor in the original stock was fortuitous, and that it was indeed selection based on tested birds that was effective in establishing the N-S line.

TABLE VII.—*Comparison of Non-Susceptible and Cross.*

| Sire. | Dam. | Tumour response. 4. | 3. | 2. | 1. | R. | 0. | Total population. |
|---|---|---|---|---|---|---|---|---|
| "N-S" | "N-S" | .. | .. | 1 | 8 | 15 | 3 | 27 |
| "N" | "N-S" | 6 | 10 | 9 | 27 | 4 | 1 | 57 |

It is difficult to make a comparison between the N-S line and material used by other workers for, in contrast to mice, very little attention has been paid in the past to the study of the genetics of cancer susceptibility in fowls. The proportions of birds reported as resistant to inoculation with Rous No. 1 sarcoma virus varies from 40 per cent (Fischer, 1927) to 4–5 per cent (Gye and Purdy, 1931). These figures may be complicated, however, by different rates of spontaneous sarcoma occurring in the population, either causing "natural" or "induced" antibodies to be formed in the tested animal. This complication is probably absent at 6 weeks (the age used here) as no antibodies are present at this stage (Amies, 1937; Andrewes, 1939; Carr, 1943*b*), though they occur in younger and older chicks. Resistance also varies with the age of the bird (Duran-Reynals, 1940; Rous and Murphy, 1914; Carr, 1943*b*), so a standard of age for testing is requisite for comparison.

In the selection experiment for high and low susceptibility to Jungherr sarcoma, carried out by Cole (1941) for example, a resistant line with only 12 per cent susceptibility was obtained, but it was found impossible to raise the high incidence line much above the original 70 per cent; the cause of this may have been acquired rather than a genetic immunity, since transmission from the immune female to the chick via the egg-yolk (Andrewes, 1939; Carr, 1944) cannot be excluded, for progeny were tested at 7–18 days, a time when yolk antibody is possibly still present in the chick. Acquired immunity was ruled out as a cause of resistance of the N-S line.

Perhaps the most useful comparison which can be made is that between the present analysis of responses of the Edinburgh flock to the filtrable tumour Rous No. 1, and those of birds from the same stock to chemical tumour inducing material as demonstrated in the work of Dr. Peacock, a report on which has been published (Greenwood and Peacock, 1945). The data, which cover the behaviour of 4 lines for the 10-year period from 1935–1944, are interesting both in their points of similarity and dissimilarity to the material already discussed: They were classified only into positive and negative groups, but the Non-moult line again showed itself the most resistant to chemically induced tumours with a susceptibility of about 30 per cent, while Breeding and Large Egg gave a figure close to 40 per cent and Intensity had the highest susceptibility with 60 per cent positives. Since the early analysis of Rous data places Breeding as highest in order of susceptibility, this difference in rank order might reflect inconsistency in the relative responses of the two lines to the different types of tumour, but it appears more likely that with continued inbreeding the Intensity line has chanced to become more susceptible. Belief that this is so has led to the majority of chicks supplied to cancer workers being mainly from this line or its crosses.

A clear difference in response to two chemically induced tumours was, however, established in Peacock's material, where the Breeding line gave a greatly increased response to a second tumour as compared with the one used originally.

The most striking point of difference between the filterable and induced tumours was the distinct seasonal swing in susceptibility exhibited by the latter. This seems to indicate that though the Non-moult line was most resistant to both types of tumour, the factors operating in respect of the two cannot be entirely the same.

### SUMMARY.

An investigation of the differing response to tumour inoculations of the inbred lines of Brown Leghorn fowl maintained at the Poultry Research Centre, Edinburgh, has been in progress for a number of years. Annual losses from spontaneous neoplastic and allied conditions are extremely low. When most of the inbred lines were tested it was found that one in particular showed an obviously high resistance to the production of fatal cancer by the Rous No. 1 virus.

The distribution of the responses in groups of chicks from individual matings makes an interpretation based on a simple genetic situation unlikely, and suggests considerable variation to non-genetic causes.

A preliminary test indicated the probability that a particular male mated to fairly close relatives would enable a non-susceptible line to be established. Breeding from his progeny showing complete lack of tumour growth resulted in resistant offspring, and subsequent generations gave only minor variations.

The particular inbred group from which the line non-susceptible to Rous No. 1 virus has been derived had shown itself earlier to be the most resistant to the transplantation of chemically induced tumours.

### REFERENCES.

Amies, C. R.—(1937) *J. Path. Bact.*, **44**, 141.
Andrewes, C. H.—(1939) *Ibid.*, **48**, 225.
Bagg, H. J.—(1936) *Amer. J. Cancer*, **26**, 69.
Carr, J. G.—(1942) *Brit. J. exp. Path.*, **23**, 339.—(1943*a*) *Ibid.*, **24**, 127.—(1943*b*) *Ibid.*, **24**, 133.—(1944) *Proc. Roy. Soc. Edinb.*, B, **62**, 54.
Cole, R. K.—(1941) *Cancer Res.*, **1**, 714.
Duran-Reynals, F.—(1940) *Yale J. Biol. Med.*, **13**, 61.
Falin, L. I., and Gromzewa, K. E.—(1939) *Amer. J. Cancer*, **36**, 223.
Fischer, A.—(1927) *Z. Krebsforsch.*, **24**, 580.
Goss, L. J.—(1940) *Cornell Vet.*, **30**, 75.
Greenwood, A. W., and Peacock, P. R. —(1945) *Brit. J. exp. Path.*, **26**, 357.
Gye, W. E., and Purdy, W. J.—(1931) 'The Cause of Cancer.' London (Cassell).
Michaelovsky, I.—(1928) *Virchow's Arch.*, **267**, 27.
Murphy, J. B., and Sturm, E.—(1941) *Cancer Res.*, **1**, 477.
Peacock, P. R.—(1935) *Amer. J. Cancer*, **25**, 37.
Rous, P., and Murphy, J. B.—(1914) *J. exp. Biol.*, **19**, 52.